# IMPACT ON PULMONARY FUNCTION AFTER SPINAL FUSION IN CONGENITAL SCOLIOSIS

**DOI:** 10.1590/1413-785220253302e284920

**Published:** 2025-06-02

**Authors:** José Alberto Alves Oliveira, Rogério dos Reis Visconti, Antônio Eulálio Pedrosa Araújo, Paulo Goberlânio de Barros Silva, Luis Eduardo Carelli, José Roberto Lapa e Silva

**Affiliations:** 1 Universidade Federal do Rio de Janeiro, Faculdade de Medicina, Programa de Pós-graduação em Medicina Clínica, Rio de Janeiro, RJ, Brazil.; 2 Instituto Nacional de Ortopedia e Traumatologia Jamil Haddad, Serviço de Cirurgia da Coluna Vertebral, Rio de Janeiro, RJ, Brazil.; 3 Instituto do Câncer do Ceará, Hospital Haroldo Juaçaba, Laboratório de Biologia Molecular, Fortaleza, CE, Brazil.

**Keywords:** Scoliosis, Respiratory Function Tests, Vital Capacity, Forced Expiratory Volume, Spinal Fusion, Escoliose, Testes de Função Respiratória, Capacidade Vital Forçada, Volume Expiratório Forçado, Fusão Vertebral

## Abstract

**Purpose:**

To evaluate the effect, in long-term postoperative follow-up (more than 15 years), of combined spinal fusion (anterior and posterior) and only posterior on the pulmonary function of patients with congenital scoliosis.

**Methods:**

Case series with five patients, operated on from 03/1997 to 12/2009, groups: dual approach with anterior arthrodesis through thoracotomy versus only posterior arthrodesis. Data processed in SPSS 20.0. Comparison of means (Student's t-test and Anova, or Mann-Whitney and Kruskal-Wallis/Dunn) with p = 0.05.

**Results:**

There was no difference in the absolute and predicted percentage values of pulmonary function FVC (Forced Vital Capacity) and FEV1 (Forced Expiratory Volume in 1 Second), Cobb of the main thoracic curve and thoracic kyphosis between the groups, in the preoperative and last follow-up (p>0.05).

**Conclusion:**

There were no significant differences in preoperative and postoperative pulmonary function parameters between the groups, after an average follow-up of 17 years, and there were also no significant differences in relation to the radiographic variables evaluated. ***Level of evidence IV; Serie Cases.***

## RESUMO

**Objetivos:**

Avaliar o efeito, em acompanhamento pós-operatório de longo prazo (maior que 15 anos), da fusão vertebral combinada (anterior e posterior) e somente posterior sobre a função pulmonar de pacientes com escoliose congênita.

**Materiais e Métodos:**

série de casos com cinco pacientes, operados de 03/1997 a 12/2009, grupos: artrodese anterior por toracotomia mais artrodese posterior versus somente artrodese posterior. Dados processados no SPSS 20.0. Comparação das médias (teste t de Student e Anova, ou Mann-Whitney e Kruskal-Wallis/Dunn) com p de 0,05.

**Resultados:**

sem diferença dos valores absolutos e percentuais previstos da função pulmonar: CVF (Capacidade Vital Forçada) e VEF1 (Volume Expiratório Forçado no 10 Segundo), do Cobb da curva torácica principal e da cifose torácica entre os grupos, no pré-operatório e pós-operatório (p>0,05).

**Conclusões:**

não se visualizou diferenças significativas dos parâmetros pré-operatórios e pós-operatórios da função pulmonar entre os grupos, após tempo de seguimento em média de 17 anos, não havendo também em relação às variáveis radiográficas avaliadas. ***Nível de evidência: IV Série de Casos.***

## INTRODUCTION

Lung development is characterized by the growth of new alveoli until 5 to 8 years of age, but thoracic development and lung function continue until 17 to 23 years of age, depending on variables such as sex and growth speed, with a reduction from 35 years old.^1^

The etiology of scoliosis is an important risk factor for the development of postoperative pulmonary complications, as well as the difficulty in recovering lung function after correction of the deformity.^2^

Some authors report that the minimum value of 65% for forced vital capacity (FVC) and forced expiratory volume in one second (FEV1), as, above this value, there is no impairment of lung function in patients with congenital scoliosis in adult life.^3^

There are few data on long-term follow-up, longer than 10 years, of changes in respiratory function in patients after surgery for scoliosis, especially for cases that are not idiopathic. As a result, this research aims to evaluate the impact of combined (anterior and posterior) and posterior-only spinal fusion on pulmonary function in patients with congenital scoliosis in long postoperative follow-up.

## MATERIAL AND METHODS

A series case study was carried a spinal surgery reference center to assess the lung function of 168 patients with scoliosis who underwent surgery from 1997 to 2009. The inclusion criteria were as follows: patients of both sexes with congenital scoliosis who underwent instrumented spinal fusion only with 3rd generation implants (hooks and pedicle screws or only pedicle screws) without thoracoplasty and/or halo traction. The exclusion criteria were a history of heart disease, pulmonary illnesses, infection, cognitive changes that influenced the understanding of the tests, and inability to perform the proposed evaluation. Eligible patients underwent an assessment during the last follow-up period with radiography and spirometry tests.

The information was obtained, after approval by the Institutional Ethics and Research Committee (06671719.9.0000.5257 and 06671719.9.3001.5273) with signed written informed consent, through medical records. During last follow-up, patients underwent pulmonary function tests (PFT) and radiographs (in anteroposterior and lateral), which were reviewed by two independent physicians who were not involved in the study. The same levels of the final vertebrae were used to calculate the preoperative and postoperative Cobb angle. The following data were collected: gender; age (at diagnosis and surgery); corrected height by wingspan; body mass index (BMI); type of approach (dual or only-posterior); duration of surgery (minutes); estimated blood loss (cc.); follow-up time until last postoperative appointment (years); pulmonary and implant-related complications, preoperative and postoperative Cobb angle of the main thoracic curve and thoracic kyphosis (T5 to T12); number of instrumented levels; absolute and predicted percentages values of Forced Vital Capacity (FVC) and Forced Expiratory Volume in the 1st second (FEV1).

Pulmonary function tests were performed with patients sitting. No patient reported a history of smoking. At least three acceptable curves were obtained, two of which were reproducible, with parameters classified according to ATS/ERS 2005 guidelines.^4^

The patients were divided into two groups: thoracotomy and posterior arthrodesis versus only posterior arthrodesis. The option for a dual approach was based on the surgical team's experience with the technique and on radiographic criteria (rigid curves, flexibility less than 40% and/or curves greater than 80°). The procedure was carried out in two stages. Interbody spinal fusion was performed during the anterior approach. In the posterior approach, PCOs (Posterior Column Osteotomies) were performed at three to four levels. During all procedures, the awakening test was used, as there was no intraoperative neurophysiological assessment with somatosensory and motor evoked potentials at the institution during this period.

The postoperative immediate pulmonary complications identified were: the presence of pleural effusion, atelectasis and respiratory failure. Regarding implant-related complications no were recorded from the immediate postoperative period until the last postoperative evaluation.

### Statistical analysis

Quantitative data were expressed as mean and standard deviation, subjected to the Kolmogorov-Smirnov normality test and compared between groups using Student's t or ANOVA/Bonferroni tests (parametric data) and Mann-Whitney or Kruskal-Wallis/ Dunn (non-parametric data) and intragroup analysis included the paired t (parametric data) and Wilcoxon (non-parametric data) tests. Categorical data were expressed as absolute and percentage frequencies and associated using Fisher's exact or Pearson's chi-square tests. All analyses were performed in SPSS version 20.0 for Windows, adopting a confidence level of 95%.

## RESULTS

This study included an initial sample of 22 patients eligible for the study obtained after meeting the inclusion and exclusion criteria, and 5 patients with spirometry at the last follow-up appointment. It was verified, when analyzing the preoperative values of clinical and radiographic variables, as well as the pulmonary function test of patients with congenital scoliosis with and without follow-up, that there were no significant differences between them (Table 1). It was found that there was no significant difference between patients undergoing combined arthrodesis and those with only posterior arthrodesis in relation to the variables: age at surgery, sex, preoperative corrected height by wingspan, preoperative BMI, fused levels, estimated blood loss and follow-up time until last postoperative appointment, which was approximately 19 years in the posterior approach group. Regarding surgical time, although surgeries with a dual approach (anterior and posterior) lasted longer, this difference was not significant (Table 2).

**Table 1:** Comparison between preoperative clinical and radiographic parameters of patients with and without follow-up.

	Follow-up		
	Yes	No	P
Age at last preoperative PFT (yrs)	13.2±3.1	15.1±6.1	0.515^a^
Gender (F/M)	4/1	10/7	0.613^b^
Corrected height by wingspan (cm)	149.2±9.2	147.4±17.9	0.834^a^
BMI	15.9±3.6	18.2±4.2	0.302^a^
FVC (L)	1.6±0.7	1.6±0.8	0.973^a^
% FVC	59.0±20.7	59.7±19.7	0.945^a^
FEV1 (L)	1.4±0.6	1.4±0.7	0.950^a^
% FEV1	57.8±18.6	58.0±19.9	0.984^a^
Cobb angle of main thoracic curve (0)	77.0±48.4	68.6±19.1	0.619^a^

* p<0,05,

a Student's T Test;

b Fisher's Exact Test. FVC, Forced Vital Capacity; FEV1, Forced Expiratory Volume in 1Second.

**Table 2:** Comparison of preoperative clinical and surgical parameters between different procedures.

	Surgery		
	Thoracotomy and posterior arthrodesis	Posterior arthrodesis	P
Age at surgery (yrs)	13.3±3.2	13.5±3.5	0.960^a^
Age at last follow-up (yrs)	31.8 ± 2.5	34.2 ± 4.2	0.48^a^
Gender (M/F)			0.40^b^
Male	3 (100.0%)	1 (50.0%)	
Female	0 (0.0%)	1 (50.0%)	
Corrected height by wingspan preoperative (cm)	146.7±6.7	153.0±14.1	0.530^a^
BMI preoperative	15.6±4.7	16.5±2.6	0.812^a^
Fused Levels	13.3±2.5	5.5±3.5	0.059^a^
Estimated Blood Loss (cc.)	2013.0±1345.5	882.0±138.6	0.343^a^
Surgical Time (min.)	491.7±235.3	227.5±3.5	0.229^a^
% FVC Preoperative			
<= 50%	2 (66.7%)	0 (0.0%)	0.082^b^
50-65%	0 (0.0%)	2 (100.0%)	
65-80%	1 (33.3%)	0 (0.0%)	
80-100%	0 (0.0%)	0 (0.0%)	
% FEV1 Preoperative			
<35%	0 (0.0%)	0 (0.0%)	0.172^b^
35 - 49%	2 (66.7%)	0 (0.0%)	
50-59%	0 (0.0%)	1 (50.0%)	
60 - 69%	0 (0.0%)	1 (50.0%)	
>70%	1 (33.3%)	0 (0.0%)	
Last Follow-up (yrs)	16.8±5.0	18.9±2.8	0.628^a^

* p<0,05,

a Student's T Test;

b Fisher's Exact Test. FVC, Forced Vital Capacity; FEV1, Forced Expiratory Volume in 1Second.

When evaluating the performance of preoperative pulmonary function between the groups, it can be seen that the majority of patients undergoing the dual approach have lower values of the FVC and the FEV1 in relation to the group with the only posterior approach, however, this difference was not significant (Table 2).

During the comparison between the groups regarding certain absolute and predicted percentages parameters of pulmonary function, as well as radiographic variables, it was noted that there were no significant differences between preoperative and postoperative values (Table 3).

**Table 3:** Analysis of pulmonary and radiographic parameters between different surgeries.

	Procedure		
	Thoracotomy and posterior arthrodesis	Posterior arthrodesis	P
**FVC (L)**			
Preoperative	1.4±0.6	2.0±0.8	0.417^a^
Postoperative	1.8±1.4	2.6±0.4	0.520^a^
P	0.468^b^	0.286^b^	
Postoperative – Preoperative	0.4±0.8	0.6±0.4	0.795^a^
**FEV1(L)**			
Preoperative	1.3±0.4	1.6±0.8	0.539^a^
Postoperative	1.5±0.9	2.1±0.4	0.418^a^
P	0.548^b^	0.353^b^	
Postoperative – Preoperative	0.2±0.5	0.5±0.4	0.542^a^
**% FVC**			
Preoperative	56.0±28.6	63.5±3.5	0.749^a^
Postoperative	55.7±33.4	73.5±3.5	0.526^a^
P	0.937^b^	0.295^b^	
Postoperative – Preoperative	-0.3±6.5	10.0±7.1	0.190^a^
**% FEV1**			
Preoperative	56.7±25.1	59.5±10.6	0.894^a^
Postoperative	80.0±35.6	72.0±0.0	0.783^a^
P	0.476^b^	0.344b	
Postoperative – Preoperative	23.3±46.5	12.5±10.6	0.778^a^
**Cobb angle of main thoracic curve (0)**			
Preoperative	99.0±53.4	44.0±5.7	0.262^a^
Postoperative	52.0±23.5	30.5±0.7	0.308^a^
P	0.181^b^	0.161^b^	
Postoperative – Preoperative	-47.0±40.3	-13.5±4.9	0.347^a^
**Kyphosis Thoracic (T5 to T12) (0)**			
Preoperative	53.3±14.4	20.0±0.0	0.184^a^
Postoperative	52.3±17.6	22.5±3.5	0.110^a^
P	0.923^b^	1.000^b^	
Postoperative – Preoperative	-1.0±15.9	+2.5±0.0	0.961^a^

* p<0,05,

a Student's T Test;

b Paired T Test. FVC, Forced Vital Capacity; FEV1, Forced Expiratory Volume in 1Second.

In the analysis of the Odds Ratio between the type of surgery versus pulmonary complications, no greater chance of these was identified to the detriment of the surgical approach used, whether combined (anterior and posterior), or only posterior (Table 4).

**Table 4:** Analysis of Odds Ratio between the postoperative pulmonary complications and the type of surgery.

	Pulmonary Complications			
	Yes	No	P	OR (CI95%)
Surgery			1.00	2.00 (0.75-5.34)
Thoracotomy and posterior arthrodesis	2 (100.0%)	2 (66.7%)		
Posterior arthrodesis	0 (0.0%)	1 (33.3%)		

* p<0,05, Fisher's Exact Test. OR = Odds Ratio; CI95% = Confidence interval 95%.

Pulmonary complications in the two patients who underwent dual approach were atelectasis and respiratory failure in one patient, pleural effusion and respiratory failure in the other.

## DISCUSSION

Regarding the assessment of pulmonary function in patients with congenital scoliosis undergoing surgery, the present study compared the preoperative results and those of the last follow-up (greater than 15 years) between the combined approach and the posterior-only approach with materials of 3rd generation.

As reported in a study by Xue et al., patients who had FVC and FEV1 values above 65% would not have impaired lung function during adult life. On the other hand, those with clinically relevant pulmonary involvement have a lower BMI and a higher Cobb angle, among other characteristics.^3^

In the current research, in both groups, the preoperative mean percentage predicted values of %FVC and %FEV1 were less than 65% in patients aged around 13 years at the time of surgery. In the follow-up time until last postoperative appointment, an increase in %FEV1 values was noticed for both groups and %FVC for the posterior arthrodesis group, although without significant diferences. In another study, which analyzed postoperative pulmonary function parameters in patients with congenital scoliosis undergoing arthrodesis, three by posterior approach and seven by combined approach, before the age of 10, an average FVC% and FEV1% were obtained around 64%. Furthermore, early surgery did not produce good radiographic results (Cobb of the main thoracic curve of 41.6°±19.2°), after seven years of follow-up.^5^

In the present series, patients who underwent the dual approach compared to the posterior approach presented, respectively, the following preoperative and postoperative Cobb angle values of the main thoracic curve (90°± 53.4°→52°± 23.5°) and (44° ± 5.7°→ 30.5° ± 0.7°). This, after a smaller number of fused levels in the posterior arthrodesis group compared to the combined (5.5 ± 3.5 versus 13.3 ± 2.5).

Other authors, when evaluating changes in lung function in patients with congenital scoliosis undergoing combined and posterior approaches, found a postoperative magnitude of the main thoracic curve of 47.6° ± 27.4° and FVC% of 68% with a mean follow-up 7.6 years ± 4.2 years. In patients with a vital capacity of less than 50% in the postoperative period, three operated by dual approach and two operated only by posterior approach, complications were pseudarthrosis in one patient and surgical wound infection in another.^6^ In the present study, the variation of Cobb angles coronal and sagittal (Kyphosis T5-T12) in the dual approach and posterior-only groups was, respectively, coronal (-47.0° ± 40.3° / 13.5° ± 5.0 °), sagittal (-1.0° ± 15.9° /2.50° ± 0°), but without significant differences. Pulmonary complications were only seen in the group that performed the dual approach, with one patient having atelectasis and respiratory failure, undergoing orotracheal intubation and antibiotic therapy; in another, pleural effusion and respiratory failure, chest drainage and orotracheal intubation were performed. It should be noted that both progressed well clinically after the measurements taken. There was no greater chance of pulmonary complications in these patients, considering the type of approach used, whether combined or only posterior. In addition, there are no records of complications related to implants.

The present research's strong point is that it is one of the few studies, if not one of the only ones, that compares preoperative and postoperative pulmonary function parameters in patients with congenital scoliosis, submitted to the combined approach versus the only posterior approach with long follow-up (greater than 15 years). However, caution must be taken when interpreting the results and in their external validation, due to the inherent limitations of this research.

While the limitations can be attributed to the fact that the study is retrospective; have been carried out in a single center; present a small sample; and not having evaluated the effect of skeletal traction, vertebral osteotomies and thoracoplasty on pulmonary function.

## CONCLUSIONS

When evaluating the effect of combined spinal fusion (anterior and posterior) and only posterior on the pulmonary function of patients with congenital scoliosis, in long-term follow-up (greater than 15 years), it was found that there was no difference in the absolute and percentages predicted values of FVC and FEV1 between the groups, in the last follow-up. Furthermore, considering that %FVC and %FEV1 are more appropriate for comparing preoperative and postoperative values for a given surgical approach, it is suggested that the dual approach did not result in worse pulmonary involvement compared to the posterior-only approach.

## References

[1] Ruiz-Juretschke C, Pizones J, Delfino R, Sánchez-Mariscal F, Zúñiga L, Izquierdo E (2017). Long-term Pulmonary Function After Open Anterior Thoracolumbar Surgery in Thoracolumbar/Lumbar Idiopathic Adolescent Scoliosis. Spine (Phila Pa 1976).

[2] Payo J, Perez-Grueso FS, Fernandez-Baillo N, Garcia A (2009). Severe restrictive lung disease and vertebral surgery in a pediatric population. Eur Spine J.

[3] Xue X, Shen J, Zhang J, Zhao H, Li S, Wang Y (2015). An analysis of thoracic cage deformities and pulmonary function tests in congenital scoliosis. Eur Spine J.

[4] Pellegrino R., Viegi G., Brusasco V., Crapo R.O., Burgos F., Casaburi R. (2005). Interpretative strategies for lung function tests. European Respiratory Journal.

[5] Vitale MG, Matsumoto H, Bye MR, Gomez JA, Booker WA, Hyman JE (2008). A retrospective cohort study of pulmonary function, radiographic measures, and quality of life in children with congenital scoliosis: an evaluation of patient outcomes after early spinal fusion. Spine (Phila Pa 1976).

[6] Day GA, Upadhyay SS, Ho EK, Leong JC, Ip M (1994). Pulmonary functions in congenital scoliosis. Spine (Phila Pa 1976).

